# A p.(Glu809Lys) Mutation in the WFS1 Gene Associated with Wolfram-like Syndrome: A Case Report

**DOI:** 10.4274/jcrpe.3021

**Published:** 2016-12-01

**Authors:** Dagmar Prochazkova, Zuzana Hruba, Petra Konecna, Jarmila Skotakova, Lenka Fajkusova

**Affiliations:** 1 Medical Faculty of Masaryk University and University Hospital Brno, Department of Pediatrics, Brno, Czech Republic; 2 Medical Faculty of Masaryk University and University Hospital Brno, Department of Internal Medicine, Division of Hematology and Oncology, Centre of Molecular Biology and Gene Therapy, Brno, Czech Republic; 3 Medical Faculty of Masaryk University and University Hospital Brno, Department of Pediatric Radiology, Brno, Czech Republic

**Keywords:** Wolfram syndrome, genotype, phenotype

## Abstract

Wolfram-like syndrome (WFSL) is a rare autosomal dominant disease characterised by congenital progressive hearing loss, diabetes mellitus, and optic atrophy. The patient was a boy with the juvenile form of diabetes mellitus and findings which clinically matched the symptoms of Wolfram syndrome. At the age of 3 1/4 years, diabetes mellitus was diagnosed in this boy who also had severe psychomotor retardation, failure to thrive, a dysmorphic face with Peters anomaly type 3 (i.e. posterior central defect with stromal opacity of the cornea, adhering stripes of the iris, and cataract with corneolenticular adhesion), congenital glaucoma, megalocornea, severe hearing impairment, a one-sided deformity of the auricle with atresia of the bony and soft external auditory canal, non-differentiable eardrum, missing os incus, hypothyreosis, and nephrocalcinosis. Molecular-genetic examinations revealed a de novo mutation p.(Glu809Lys) in the WFS1 gene. No mutations were detected in the biological parents. The mutation p.(Glu809Lys) in the WFS1 gene is associated with WFSL.

**WHAT IS ALREADY KNOWN ON THIS TOPIC?**

The Wolfram syndrome (WFS, Online Mendelian Inheritance in Man 222300), also known as the DIDMOAD syndrome (diabetes insipidus, early-onset diabetes mellitus, progressive optic atrophy, and deafness) is mostly associated with a recessive mutation in the WFS gene 1 (WFS1), rarely in the WFS2. A dominant mutation in the WFS1 gene was described in connection with sensorineural hearing loss, deafness, and optic atrophy: Wolfram-like syndrome (WFSL). Variable clinical symptoms, rare occurrence, and molecular complexity complicate the diagnosis and the genotype-phenotype correlation of the disease.

**WHAT THIS STUDY ADDS?**

The novelty of the data and their impact on the field: the p.(Glu809Lys) mutation in the WFS1 gene is associated with the occurrence of the WFSL.

## INTRODUCTION

Wolfram syndrome (WFS, OMIM 222300) is a rare genetic disease with a prevalence of cca. 1:710.000 (1). The disease is also known as the DIDMOAD syndrome (diabetes insipidus, diabetes mellitus, optic atrophy, deafness). Most of the individuals afflicted with this disease have the recessive mutation in the WFS gene 1 (WFS1, 4p16.3) (1,2) and rarely in the WFS gene 2 (WFS2) (3). A dominant mutation in the WFS1 gene was also described in connection with sensorineural hearing loss, deafness, and optic atrophy [Wolfram-like syndrome (WFSL)] (4).

## CASE REPORT

Our patient was a boy whose clinical findings matched those reported for the Wolfram syndrome. At the age of 3 1/4 years, the patient was diagnosed to have diabetes mellitus along with severe psychomotor retardation, failure to thrive, a dysmorphic face with Peters anomaly (PS) type 3 (posterior central defect with stromal opacity of the cornea, adhering stripes of the iris, and cataract with corneolenticular adhesion), congenital glaucoma, megalocornea, hypothyreosis, nephrocalcinosis, severe hearing impairment, a one-sided deformity of the auricle with atresia of the bony and soft external auditory canal, a non-differentiable eardrum, and a missing os incus. At presentation, the patient did not have optic atrophy or diabetes insipidus. The proband’s karyotype was 46,XY, 9qh+.

We performed sequencing analysis of the coding exons and adjacent intron regions of the WFS1 gene and of the B3GALTL, CYP1B1, PITX2, and PAX6 genes associated with PS. The analysis for the WFS1 gene was complemented by multiple ligation-dependent probe amplification (SALSA MLPA P163 GJB-WFS1, MRC-Holland) identifying potential deletions/duplications. Apart from standard polymorphisms, the only potentially causal mutation was found to be a heterozygous missense mutation in the WFS1 gene, namely, c.2425G>A, p.(Glu809Lys). This mutation was analysed in the proband’s family, namely, in both parents and siblings of the proband-twin B. The mutation was not detected in any of the family members and the final diagnosis was WFSL caused by a de novo mutation c.2425G>A, p.(Glu809Lys). This mutation localised in the exon 8 of WFS1 was not reported in databases (http://databases.lovd.nl/whole_genome/genes or http://exac.broadinstitute.org/) and was described as a likely pathogenic finding in http://www.ncbi.nlm.nih.gov/clinvar/variation/215413/. In silico analysis of this mutation using prediction programs PolyPhen-2 and SIFT showed probable damaging and damaging effects, respectively.

## DISCUSSION

In 2014, Matsunaga et al (1) described a proband with WFS and c.2425G>A, p.(Glu809Lys) mutation in the WFS1 gene. The patient suffered from diabetes mellitus, optic atrophy, deafness, and mental disorder. In the same year, Lee et al (5) mentioned a proband with c.2425G>A, p.(Glu809Lys) mutation in the WFS1 gene. The proband suffered from cataract, hypotonia, sensorineural deafness, and diabetes mellitus. The second causal mutation was not determined in either patient. Follow-up molecular genetic examinations of the family members had apparently not been carried out in these cases. We believe that molecular genetic examination in the members of the family of a proband with one mutation in the WFS1 gene is important.

## Acknowledgments

Our thanks go to the Department of Pediatric Otorhinolaryngology University Hospital Brno and the Department of Pediatric Ophthalmology University Hospital Brno for the care of our patient.

## Ethics

Informed Consent: It was taken.

Peer-review: Externally peer-reviewed.
